# A new cold-adapted β-D-galactosidase from the Antarctic *Arthrobacter *sp. 32c – gene cloning, overexpression, purification and properties

**DOI:** 10.1186/1471-2180-9-151

**Published:** 2009-07-27

**Authors:** Piotr Hildebrandt, Marta Wanarska, Józef Kur

**Affiliations:** 1 Department of Microbiology, Chemical Faculty, Gdańsk University of Technology, Narutowicza 11/12, 80-952 Gdańsk, Poland

## Abstract

**Background:**

The development of a new cold-active β-D-galactosidases and microorganisms that efficiently ferment lactose is of high biotechnological interest, particularly for lactose removal in milk and dairy products at low temperatures and for cheese whey bioremediation processes with simultaneous bio-ethanol production.

**Results:**

In this article, we present a new β-D-galactosidase as a candidate to be applied in the above mentioned biotechnological processes. The gene encoding this β-D-galactosidase has been isolated from the genomic DNA library of Antarctic bacterium *Arthrobacter *sp. 32c, sequenced, cloned, expressed in *Escherichia coli *and *Pichia pastoris*, purified and characterized. 27 mg of β-D-galactosidase was purified from 1 L of culture with the use of an intracellular *E. coli *expression system. The protein was also produced extracellularly by *P. pastoris *in high amounts giving approximately 137 mg and 97 mg of purified enzyme from 1 L of *P. pastoris *culture for the AOX1 and a constitutive system, respectively. The enzyme was purified to electrophoretic homogeneity by using either one step- or a fast two step- procedure including protein precipitation and affinity chromatography. The enzyme was found to be active as a homotrimeric protein consisting of 695 amino acid residues in each monomer. Although, the maximum activity of the enzyme was determined at pH 6.5 and 50°C, 60% of the maximum activity of the enzyme was determined at 25°C and 15% of the maximum activity was detected at 0°C.

**Conclusion:**

The properties of *Arthrobacter *sp. 32cβ-D-galactosidase suggest that this enzyme could be useful for low-cost, industrial conversion of lactose into galactose and glucose in milk products and could be an interesting alternative for the production of ethanol from lactose-based feedstock.

## Background

Nowadays low-cost energy bio-industrial processes in biotechnology are highly desired. This has led to increased interest in the production of cold adapted enzymes. One class of such enzymes includes cold-adapted β-D-galactosidases (EC 3.2.1.23) that can find many applications in industrial biotechnology. These enzymes are capable of hydrolyzing 1,4-β-D-galactoside linkages and can sometimes catalyse the synthesis of oligosaccharides. The production of lactose-free milk and synthetic oligosaccharides like lactulose are only examples of this cutting edge enzyme class application.

Currently, commercially available β-galactosidase preparations (e.g. Lactozym – Novo Nordisk, Maxilact – DSM Food Specialties) applied for lactose hydrolysis contain *Kluyveromyces lactis *β-galactosidase naturally intracellularly biosynthesized by *K. lactis *strains. This enzyme is optimally active at approximately 50°C and displays low activity at 20°C while an ideal enzyme for treating milk should work well at 4–8°C. Besides, the latter enzyme should be optimally active at pH 6.7–6.8 and cannot be inhibited by sodium, calcium or glucose. Such β-galactosidases are still highly desired. Only several enzymes optimally hydrolyzing lactose at low temperatures have been characterized till now [1-14], however, none of them have been produced on the commercial scale. The β-galactosidases were obtained from different microbial sources, including those from *Arthrobacter *sp. [1,2,7,8,12], *Arthrobacter psychrolactophilus *[9,13]*Carnobacterium piscicola *[3], *Planococcus *sp. [4,14], *Pseudoalteromonas haloplanktis *[5], and *Pseudoalteromonas *sp. [10,11].

Additionally, in order to make progress in cheaper production of β-D-galactosidases of industrial interest, high efficiency yeast expression systems must be taken into consideration. On the other hand extracellular production must occur to allow easy and fast isolation of target protein. There are several studies in literature related to the extracellular production of the *Aspergillus niger *β-galactosidase by recombinant *Saccharomyces cerevisiae *strains [15-19], although this enzyme is mainly interesting for lactose hydrolysis in acid whey, because of their acidic pH optimum as well as their activity at elevated temperatures. The *S. cerevisiae *expression system was also used for the production of *K. lactis *β-D-galactosidase, the protein of outstanding biotechnological interest in the food industry but in this case the enzyme production was not strictly extracellular. The β-galactosidase was released into the culture medium after osmotic shock of the recombinant *S. cerevisiae *osmotic-remedial thermosensitive-autolytic mutants [20,21]. To improve the secretion of the *K. lactis *β-D-galactosidase, cytosolic in origin, the hybrid protein from this enzyme and its *A. niger *homologue, that is naturally extracellular, was constructed. The hybrid protein was active and secreted by recombinant *K. lactis *strain, but the amount of extracellular enzyme still remained low [22]. Yeast species especially designated for the production of extracellular proteins are for example *Pichia pastoris *or *Hansenula polymorpha*. There is only one recently published example of an extracellular β-galactosidase production system using *P. pastoris *as a host, however, it concerns thermostable enzyme from *Alicyclobacillus acidocaldarius *[23].

*S. cerevisiae *is usually the first choice for industrial processes involving alcoholic fermentation but this yeast is unable to metabolize lactose and, therefore, the lactose consuming yeast, *K. fragilis*, has been used in most industrial plants producing ethanol from whey [24]. The engineering of *S. cerevisiae *for lactose utilization has been addressed over the past 20 years by different strategies [25]. However, most recombinant strains obtained displayed no ideal characteristics (such as slow growth, genetic instability or problems derived from the use of glucose/galactose mixtures) or were ineffective for ethanol production [24,26,27]. There is only one published example of efficient ethanol production with a recombinant *S. cerevisiae *strain expressing the LAC4 (β-galactosidase) and LAC12 (lactose permease) genes of *K. lactis *[28]. Hence, there is still a need for *S. cerevisiae *strains producing new β-galactosidases which may appear to be an interesting alternative for the production of ethanol from lactose-based feedstock.

In this respect, here we report on a new cold-adapted β-D-galactosidase, isolated from psychrothrophic, Antarctic *Arthrobacter *sp. 32c bacterium strain, that possesses low molecular weight of 75.9 kDa of monomer and 195 kDa of native protein. In addition, the presented enzyme is active in the range of temperature 4–8°C that is suitable for milk industry applications and can be produced extracellularly on a large scale using recombinant *P. pastoris *strains cultivated either on methanol or glycerol (a cheap by-product in biodiesel industry).

## Results

### Characterisation of 32c isolate

**Figure 1:**
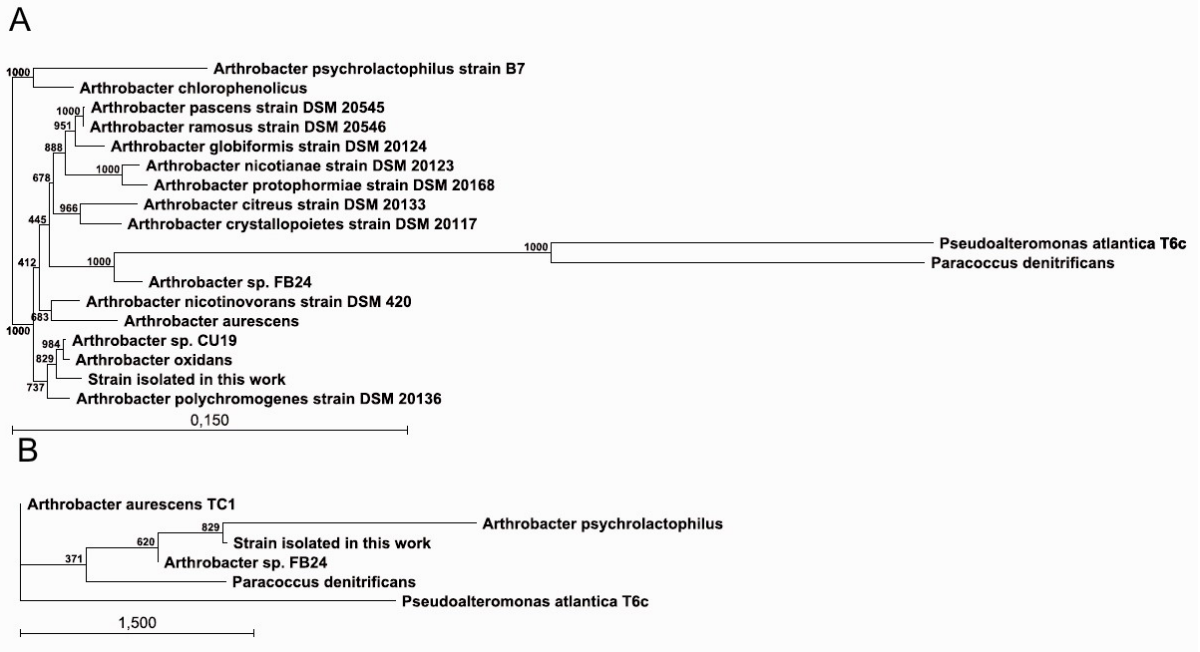
**Phylogenetic analysis of the *Arthrobacter *sp. 32c 16S rDNA sequence (A) and *Arthrobacter *sp. 32c β-D-galactosidase gene sequence (B)**. Sequences were aligned using the sequence analysis softwares: ClustalX 1.5 b and Gene-Doc 2.1.000. Phylogenetic trees were reconstructed with the PHYLIP COMPUTER PROGRAM PACKAGE, using the neighbour-joining method with genetic distances computed by using Kimura's 2-parameter mode. The scale bar indicates a genetic distance. The number shown next to each node indicates the percentage bootstrap value of 100 replicates.

Many different colonies were isolated from the Antarctic soil. One isolate, named 32c, that formed yellow colonies was chosen for further study because of its ability to hydrolyze X-Gal – the cromogenic analogue of lactose. The cells were Gram-negative rods. The optimum growth in LAS medium was observed between 25–27°C. No growth occurred at 37°C. In order to determine the ability of the selected isolate to utilize starch, milk, avicell or arabinose several plates with different substrates were prepared. It was observed that 32c strain produces enzymes of industrial interest like α-amylase, proteases and has an arabinose utilization pathway. In order to estimate the phylogenetic position of the isolate, we cloned the amplified 16S rRNA gene into pCR-Blunt vector, determined its sequence, and examined its phylogenetic relationships (Fig. 1A). The obtained sequence was deposited at GenBank with the accession no. FJ609656. An analysis of the sequence showed that it clustered with other organisms isolated from cold environments, mainly belonging to *Arthrobacter *species. The isolate formed a well-defined cluster with *A. oxidans *(98.59% sequence identity) and *A. polychromogenes *(97.86% sequence identity). Based on 16S rDNA similarity, physiological properties similar to other *Arthrobacter *strains and its presence in the Antarctic soil our isolate was classified as *Arthrobacter *sp. 32c.

### Characterisation of the β-D-galactosidase gene

The psychrotrophic *Arthrobacter *sp. 32c chromosomal library was prepared in *E. coli *TOP10F'. The plasmid pBADmycHisA was used to construct the library, and ampicillin-resistant transformants were selected and screened for the ability to hydrolyze X-Gal. Several transformants out of approximately 5,000 were selected as blue colonies on plates containing X-Gal. Restriction analysis of plasmid inserts from these transformants indicated that they had been derived from the same fragment of chromosomal DNA. Sequence data from the shortest construct, named pBADmycHisALibB32c, contained 5,099 bp insert with an open reading frame (2,085 bp) encoding protein, which shares high homology to a β-D-galactosidase (NCBI Access No. ** FJ609657**). The sequence of *Arthrobacter *sp. 32c β-D-galactosidase was analyzed and found to encode a 694 amino acid protein with a predicted mass of 76.142 kDa and a theoretical pI of 5.59. The analysis of DNA sequence upstream the *Arthrobacter *sp. 32c β-D-galactosidase gene with the promoter prediction tool (BPROM software, http://www.softberry.com) revealed a potential promoter sequence with cttaca and tacaat as -35 and -10 sequences, respectively. A putative ribosomal binding site was apparent 8 bases before the initiating methionine codon. The insert fragment and β-D-galactosidase gene had a high G+C content, 67 mol% and 66 mol%, respectively, which is typical of *Arthrobacter *species.

A comparison of the *Arthrobacter *sp. 32c β-D-galactosidase gene sequence with those from the NCBI database showed that it was most closely related to the *Arthrobacter *sp. FB24 gene (77.13% sequence identity) and to the *A. aurescens *TC1 gene (71.8% sequence identity) (Fig. 1B). The deduced amino acid sequence from *Arthrobacter *sp. 32c β-D-galactosidase gene was also used to compare with other amino acid sequences deposited in the NCBI database. The *Arthrobacter *sp. 32c β-D-galactosidase was found to be a member of the glycoside hydrolase family 42 and contained an A4 beta-galactosidase fold. The enzyme shares 84% of identity and 91% of similarity to the sequence of the *Arthrobacter *sp. FB24, 74% identity and 84% similarity to the sequence of the *Arthrobacter aurescens *TC1 and only 51% identity and 65% similarity to the sequence of the *Janibacter *sp. HTCC2649 β-D-galactosidase.

### Overexpression and purification of recombinant *Arthrobacter *sp. 32c β-D-galactosidase

**Figure 2:**
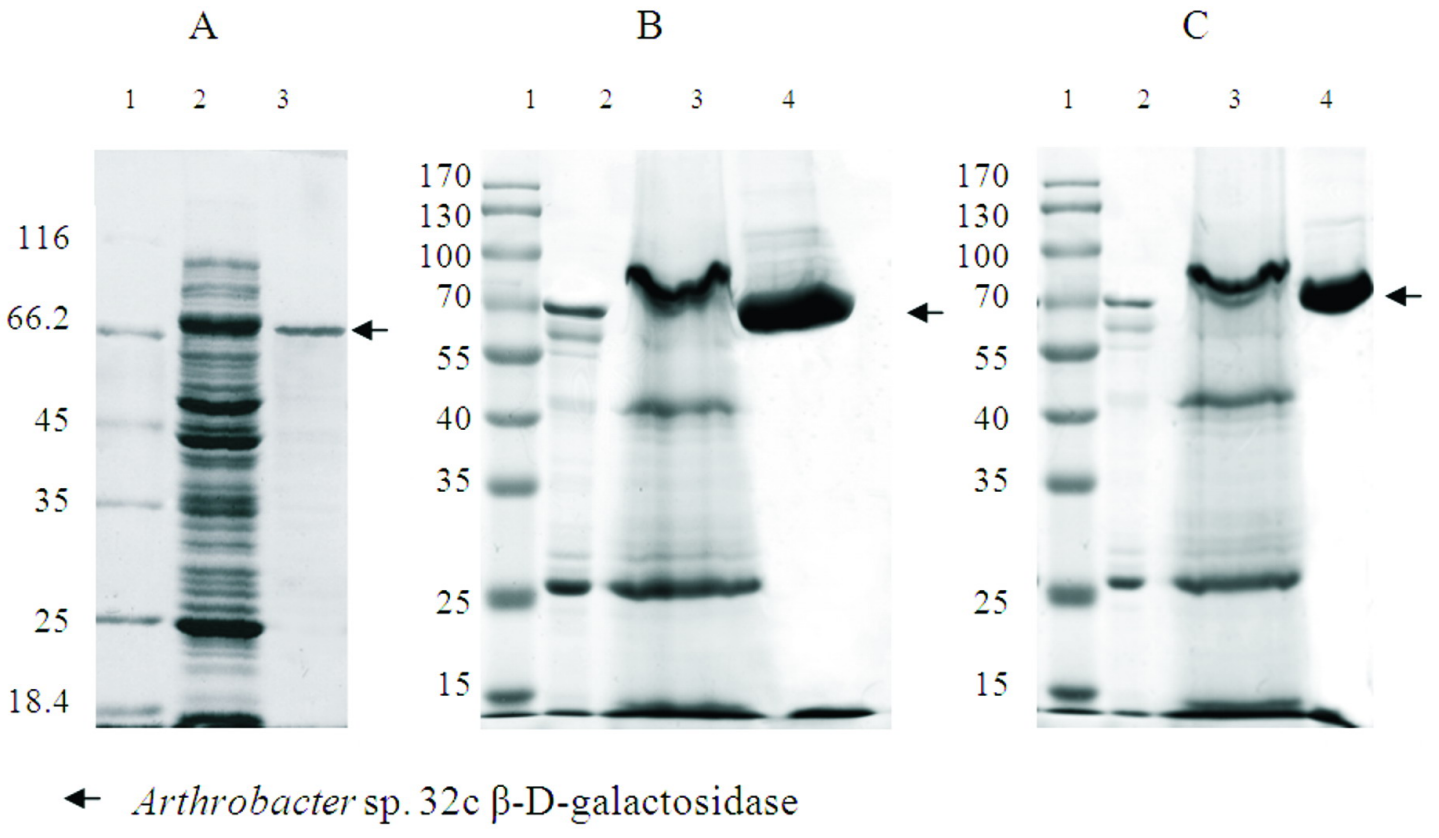
**SDS-PAGE analysis of the expression and purification steps of the *Arthrobacter *sp. 32c β-D-galactosidase expressed by *E. coli *host (A), *P. pastoris *GS115 pPICZαA-32cβ-gal methanol induced variant (B) and *P. pastoris *GS115 pGAPZαA-32cβ-gal constitutive variant (C)**. Lanes 1 – protein weight marker. Panel A: lane 2 – cell extract after expression, lane 3 – purified β-D-galactosidase after affinity chromatography. Panel B and C: lane 2 – broth after protein expression, lane 3 – protein precipitate, lane 4 – purified β-D-galactosidase after affinity chromatography.

In order to produce and investigate the biochemical properties of *Arthrobacter *sp. 32c β-D-galactosidase, we constructed bacterial and yeast expression systems. The recombinant arabinose-inducible pBAD-Myc-HisA-β-gal32c plasmid was used for the expression of the *Arthrobacter *sp. 32c β-D-galactosidase gene in *E. coli *LMG194/plysN [29]. The highest enzyme biosynthesis yields were achieved by adding arabinose to the final concentration of 0.02% w/w, at A_600 _0.5 and by further cultivation for 5 h. After purification a single protein migrating near 70 kDa was observed following sodium dodecyl sulfate-polyacrylamide gel electrophoresis and staining with Coomassie blue (Fig. 2A, lane 3). It was in good agreement with the molecular mass deduced from the nucleotide sequence (75.9 kDa). The applied overexpression system was quite efficient, giving 27 mg (Table 1) of purified β-D-galactosidase from 1 L of induced culture. The relative molecular mass of native enzyme estimated by gel filtration on a column of Superdex 200 HR 10/30, previously calibrated with protein molecular mass standards, was 195,550 Da. Hence, it is assumed that the purified *Arthrobacter *sp. 32c β-D-galactosidase is probably a trimeric protein.

**Table 1:** Purification of recombinant *Arthrobacter *sp. 32c β-D-galactosidase.

Purification step	Volume (ml)	Protein (mg)	Specific activity (U mg^-1^)	Total activity (U)	Purification (fold)	Recovery (%)
*E. coli *LMG plysN pBADMyc-HisA-32cβ-gal						

Cell extract	30	580	13.8	8004	1.0	100
Affinity chromatography	3.2	27	155.9	4209	21.0	53

*P. pastoris *GS115 pPICZαA-32cβ-gal						

Broth	1000	3400	28.7	97580	1.0	100
Protein precipitation	54	340	136.1	46274	10.0	47
Affinity chromatography	11	137	154.7	21194	24.8	22

*P. pastoris *GS115 pGAPZαA-32cβ-gal						

Broth	1000	5200	16.2	84240	1.0	100
Protein precipitation	46	450	102.7	46215	11.6	55
Affinity chromatography	10	97	153.1	14851	53.6	18

In the *P. pastoris *expression system the methanol induced and constitutive biosynthesis variants for larger scale production of the enzyme were tested. By cloning the gene in the form of translational fusion with the *S. cerevisiae *α-factor leader sequence under the control of either the methanol induced promoter AOX1 or under the constitutive promoter GAP, pPICZαA-32cβ-gal and pGAPZαA-32cβ-gal recombinant expression plasmids were constructed. *P. pastoris *GS115 strain was transformed with linearized pPICZαA-32cβ-gal or pGAPZαA-32cβ-gal plasmids. The obtained *P. pastoris *GS115 recombinant strains harbouring pGAPZαA-32cβ-gal or pPICZαA-32cβ-gal recombinant plasmids were used for extracellular production of the *Arthrobacter *sp. 32c β-D-galactosidase (Fig. 2B, lane 2 and Fig. 2C, lane 2). The applied overexpression systems were efficient, giving approximately 137 and 97 mg (Table 1) of purified β-D-galactosidase (Fig. 2B and 2C, lanes 4) from 1 L of induced culture for the AOX1 and constitutive system, respectively. Noteworthy is the fact that all attempts in extracellular expression of β-D-galactosidase from *Pseudoalteromonas *sp.22b [10,11] previously described by us did not succeed (data not shown). The corresponded β-D-galactosidase is a tetramer composed of 115 kDa subunits. All the amount of produced protein with fused secretion signal was accumulated in the cells. We also tried to produce the *Pseudoalteromonas *sp. 22b β-D-galactosidase in the form of fusion protein with other secretion sequences: PHO5 and STA2. All attempts gave negative results. It seems that molecular mass of desired recombinant protein is limited for extracellular production by *P. pastoris *host.

### Characterization of *Arthrobacter *sp. 32c β-D-galactosidase

**Figure 3:**
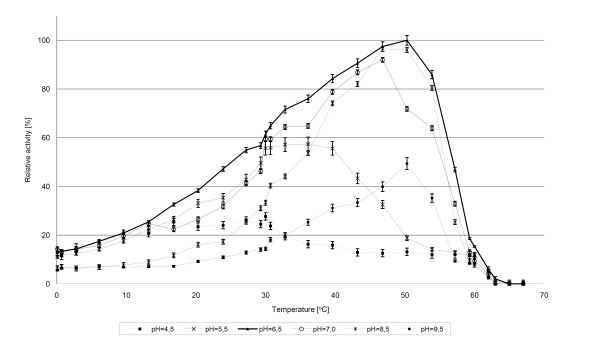
**Effect of temperature on activity of recombinant *Arthrobacter *sp. 32c β-D-galactosidase at pH range from 4.5 to 9.5**.

The temperature profiles of the hydrolytic activity of the recombinant *Arthrobacter *sp. 32c β-D-galactosidase showed that the highest specific activity with ONPG was at 50°C (155 U/mg). Lowering or raising temperature from 50°C resulted in the reduction of β-D-galactosidaseactivity. Recombinant β-D-galactosidase exhibited 15% of the maximum activity even at 0°C and approximately 60% at 25°C (Fig. 3). In order to determine the optimum pH for recombinant β-D-galactosidase, we measured the enzyme activity at various pH values (pH 4.5–9.5) at 0–70°C, using ONPG as a substrate. β-D-galactosidase exhibited maximum activity in pH 6.5 and over 90% of its maximum activity in the pH range of 6.5–8.5 (Fig. 3).

To examine the possible metal ion requirements, the enzyme preparation was treated with EDTA to remove metal ions. No activity was lost during treatment with 100 mM EDTA after 2 h. The activity was not considerably affected by metal ions (5 mM): Na^+^, K^+^, Mg^2+^, Co^2+^, Ca^2+^. The enzyme activity was completely inhibited by Cu^2+ ^or Zn^2+ ^(5 mM) and was strongly inhibited by Mn^2+ ^(11%), Fe^2+^(25%) and Ni^2+ ^(38%) in comparison to the activity of the enzyme in the absence of cations (100%) (Table 2). The activity of the β-D-galactosidase was not considerably affected by ditiothreitol, β-mercaptoethanol, and L-cysteine, whereas reduced glutathione almost completely inactivated the enzyme (Table 3). The examination of the ethanol influence on the *Arthrobacter *sp. 32c β-D-galactosidaseactivity with ONPG as the substrate shows that addition of ethanol up to 20% still slightly stimulates the enzyme activity (Table 4). The relative enzyme activity was increasing up to 120% in the presence of 8% v/v ethanol at pH 5.5.

**Table 2:** Effects of metal ions on *Arthrobacter *sp. 32c β-D-galactosidase activity.

Metal ion	Relative activity [%]
None	100
Na^+^	97 ± 3
K^+^	100 ± 2
Ni^2+^	38 ± 4
Mg^2+^	90 ± 2
Fe^2+^	25 ± 2
Co^2+^	87 ± 3
Cu^2+^	0 ± 0
Mn^2+^	11 ± 2
Zn^2+^	0 ± 0
Ca^2+^	88 ± 2

**Table 3:** Effects of thiol compounds on recombinant *Arthrobacter *sp. 32c β-D-galactosidase activity.

Compound	Relative activity [%]
None	100
2-mercaptoethanol	92 ± 4
DTT	96 ± 2
Glutathione reduced	6 ± 3
L-cystein	95 ± 2

**Table 4:** Effect of ethanol concentration on recombinant *Arthrobacter *sp. 32c β-D-galactosidase activity.

Ethanol [% v/v]	Relative activity [%] pH 5.5	Relative activity [%] pH 6.5
0	100	100
1	109 ± 2.0	102 ± 2.4
2	111 ± 2.2	107 ± 3.0
4	114 ± 2.7	109 ± 2.6
6	116 ± 2.5	110 ± 2.4
8	120 ± 2.1	111 ± 2.4
10	119 ± 2.3	109 ± 2.5
12	117 ± 1.9	107 ± 2.6
14	109 ± 2.2	105 ± 2.4
16	108 ± 2.1	103 ± 2.5
18	105 ± 2.7	102 ± 2.7
20	103 ± 2.9	101 ± 3.1

A study of the substrate specificity of the *Arthrobacter *sp. 32c β-D-galactosidase was performed with the use of various chromogenic nitrophenyl analogues. The recombinant *Arthrobacter *sp. 32c β-D-galactosidase displayed four times higher level of activity with PNPG (*p*-nitrophenyl-β-D-galactopyranoside) than with ONPG (*o*-nitrophenyl-β-D-galactopyranoside) as substrate. The activities with PNPGlu (*p*-nitrophenyl-β-D-glucopyranoside) and ONPGlu (*o*-nitrophenyl-β-D-glucopyranoside) were significantly lower with only 1.4% and 0.5% of the activity with ONPG, respectively.

In order to further characterize the biochemical properties of the enzyme the highest specific activity k_cat_, the K_M _values and the catalysis efficiency k_cat_/K_M _in reaction with ONPG and lactose were calculated. The highest observed specific activity with ONPG was 212.4 s^-1 ^at 50°C. The half saturation coefficient (K_M_) was highest at 10°C (5.75 mM), decreased to 2.62 mM at 50°C and rose again to 5.11 mM at 55°C. The highest catalysis efficiency was achieved at 50°C (81.7 s^-1^mM^-1^). The same kinetic parameters were also determined with lactose (Table 5). Hereby the half saturation coefficient was significantly higher, the reaction velocity constant was significantly lower and the reaction efficiency was very low. To investigate the reason for such results another test was performed, where glucose was transformed in the reaction mixture by glucose isomerase that converted it to fructose, while galactose remained in the mixture. In this test the reaction efficiency was significantly higher and over 30% from the 5% w/v of lactose was hydrolysed to glucose and galactose for 12 hours and over 75% of the lactose was found to be hydrolysed after 72 hours. These results were similar to another test where the recombinant *P. pastoris *strain extracellularly producing *Arthrobacter *sp. 32c β-D-galactosidase (pGAPZαA-32cβ-gal) was cultivated on lactose containing broth. It seems obvious that *Arthrobacter *sp. 32c β-D-galactosidase is inhibited by glucose. Nevertheless this shows that the enzyme might successfully catalyse the conversion of lactose to corresponding monocarbohydrates in a fermentation broth where glucose is consumed by cells of the fermenting strain.

**Table 5:** Kinetic parameters of *Arthrobacter *sp. 32c β-D-galactosidase.

Substrate	Temperature [°C]	K_m _[mM]	k_cat _[s^-1^]	k_cat_/K_m _[s^-1^mM^-1^]
ONPG	10	5.75 ± 0.34	52.4 ± 0.72	9.12 ± 0.71
	20	4.86 ± 0.37	81.0 ± 1.03	16.67 ± 1,60
	30	3.46 ± 0.29	123.9 ± 1.21	35.81 ± 3.66
	40	3.15 ± 0.27	169.9 ± 1.44	53.92 ± 5.56
	50	2.62 ± 0.21	212.4 ± 1.67	81.07 ± 7.76
	55	5.11 ± 0.32	71.2 ± 0.98	13.93 ± 1.14

lactose	10	77.54 ± 1.77	1.76 ± 0.11	0.023 ± 0.002
	20	67.82 ± 1.74	2.36 ± 0.14	0.035 ± 0.003
	30	52.67 ± 1.71	4.81 ± 0.22	0.091 ± 0.007
	40	44.31 ± 1.73	5.73 ± 0.21	0.129 ± 0.010
	50	39.73 ± 1.72	6.98 ± 0.23	0.176 ± 0.014

## Discussion

The β-D-galactosidase from *Arthrobacter *sp. 32c characterized in this study has interesting industrial properties. It displays optimum activity at pH 6.5 and catalyses the hydrolysis of 1,4-β-D-galactoside linkages at pH 4.5–9.5 with high efficiency. Its optimum activity was observed at about 50°C. Nevertheless it showed over 50% of activity at pH 5.5–7.5 at 30°C and was not considerably inactivated by Ca^2+ ^ions what in fact can be of interest in industrial ethanol production from cheese whey by means of brewing *Saccharomyces cerevisiae *strains or by recombinant strains that simultaneously utilize glucose and galactose.

β-D-galactosidases naturally produced by psychrophilic microorganisms are either intracellular or expressed at low levels. In order to make progress in cheaper production of β-D-galactosidases of industrial interest, we choose highly efficient *P. pastoris *expression systems for consideration to produce enzyme extracellularly. *P. pastoris *has been successfully used many times in extracellular protein production, however, there are only several examples of cold-adapted proteins and none cold-adapted β-D-galactosidase produced by this host. We have found only one published example of *P. pastoris *extracellular β-D-galactosidase production for a thermostable enzyme from *Alicyclobacillus acidocaldarius *[23].

There are several examples of cold active β-D-galactosidases isolated from *Pseudoalteromonas *strains [5,10,11] and *Arthrobacter *strains [7-9,12,13] with molecular mass above 110 kDa of monomer and forming an active enzyme of over 300 kDa. Most of them belong to the family 42 β-D-galactosidases. However, the β-D-galactosidase belonging to family 2 obtained from the Antarctic *Arthrobacter *isolate appears to be one of the most cold-active enzymes characterized to date [8]. All of the known cold-adapted β-D-galactosidases, except two of them isolated from *Planococcus *sp. strains [4,14] and from *Arthrobacter *sp. 32c (this study), form very large oligomers and therefore are of minor interest in industrial application probably because of many problems in effective overexpression. The β-D-galactosidases isolated from psychrophilic *Planococcus *sp. strains have low molecular weight of about 75 kDa of monomer and about 155 kDa of native protein. The β-D-galactosidase isolated from *Planococcus *sp. L4 is particularly thermolabile, loosing its activity within only 10 min at 45°C [14] and therefore larger scale production of this enzyme by recombinant yeast strains cultivated at 30°C might be economically not feasible. Only the β-D-galactosidase from *Planococcus *sp. isolate SOS orange [4] displays interesting activity and might be considered in biotechnological production on a larger scale.

In comparison with known β-D-galactosidases, the *Arthrobacter *sp. 32c β-D-galactosidase is a protein with a relatively low molecular weight. Molecular sieving revealed that the active enzyme is a trimmer with a molecular weight of approximately 195 ± 5 kDa. Relatively low molecular weight of the protein did not interfere with extracellular production of the protein by *P. pastoris*. Therefore the constructed recombinant strains of *P. pastoris *may serve to produce the protein extracellularly with high efficiency and in a cheap way. The calculated production cost of 1 mg of purified β-D-galactosidase was estimated at 0.03 €.

The same *Pichia pastoris *expression systems had been unsuccessfully used for extracellular expression of previously reported β-D-galactosidase from *Pseudoalteromonas *sp. 22b [10,11]. This enzyme is much bigger than *Arthrobacter *sp. 32c β-D-galactosidase and forms a tetramer of approximately 490 kDa. It is worth noting that we have tried to secrete this enzyme with three different secretion signals (α-factor from *Saccharomyces cerevisiae*, glucoamylase STA2 from *Saccharomyces diastaticus *or phosphatase PHO5 from *S. cerevisiae*) with no success. It seems that the molecular mass of the desired recombinant protein is limited to extracellular production by *P. pastoris *host, whereas the used secretion signal is without any influence. Based on our experience with *Pichia pastoris *expression systems we assert that the larger protein the lower expression yield can be achieved.

In comparison with the known β-D-galactosidase from *Planococcus *sp. isolate SOS orange [10], β-D-galactosidase from *Arthrobacter *sp. 32c is more thermostable and it has a similar activity profile. Moreover, as shown in this study, it can be produced extracellularly in high amounts by yeast strain. The displayed activity profile of the *Arthrobacter *β-D-galactosidase, especially the activity at pH range from 5.5 to 7.5, over 50% of relative activity at 30°C and enhancement of the activity by the presence of ethanol suggest that this enzyme is compatible with the industrial process conditions for ethanol production by yeast. The construction of corresponding *S. cerevisiae *recombinant strains and fermentation tests for the production of ethanol from cheese whey by the application of this β-D-galactosidase are pending.

The *Arthrobacter *β-D-galactosidase was strongly inhibited by glucose and therefore the catalysis efficiency was very low. Removal of this product resulted in 75% hydrolysis of a solution containing 5% of lactose after 72 hours in a combined enzyme assay. These results clearly indicate that the enzyme can be used for the production of sweet lactose free milk where hydrolysis of lactose to glucose and galactose is performed by simultaneous isomerisation of glucose to fructose by glucose isomerase.

## Conclusion

In this study we present the purification and characterisation of a new β-D-galactosidase from *Arthrobacter *sp. 32c. From the sequence analyses it is obvious that the protein is a member of the family 42 β-D-galactosidases. The protein weight deduced from the 695 amino acid sequence was 75.9 kDa. Molecular sieving revealed that the active enzyme has a molecular weight of approximately 195 ± 5 kDa and therefore it is probably a trimmer. The new characterised β-D-galactosidase is of industrial interest and can be produced extracellularly in its economically feasible variant by the constructed *P. pastoris *strain.

The constructed *P. pastoris *strain may be used in co-fermentation of lactose from cheese whey by a consortium of microorganisms with industrial strains of brewing yeast *S. cerevisiae*, where the *P. pastoris *produces β-D-galactosidase in the oxygen phase and accelerates the shift between the oxidative and reductive conditions.

## Methods

### Isolation, characterisation and identification of the 32c isolate

A 5 g of Antarctic soil was dissolved in 45 ml of water containing 1% of sea salt (Sigma-Aldrich). After decantation 100 μl of the supernatant was spread out on LAS agar plates that contained 1% lactose, 0.1% pepton K, 0.1% yeast extract, 1% of marine salt, 1.5% agar and 20 μg/ml of X-gal. Pure cultures of microorganisms were isolated. One of them was found to be a producer of β-D-galactosidase and also exhibited amylolytic and proteolytic activities. This strain was primarily classified as 32c isolate and used for further analyses. The bacterium 32c was cultured in the liquid LAS medium containing 1% lactose, 1% pepton K, 0.5% yeast extract and 1% artificial sea salt at 15°C for 2 days at 150 rpm in air shaker. The temperature profile of growth was determined in the range 0–37°C, by means of stationary cultures in the LAS medium.

### 16S rDNA gene amplification

Genomic DNA from isolate 32c was used as a template to amplify 16S rDNA gene using primers: 16S For 5' AGAGTTTGATCCTGGCTCAG 3' and 16S Rev 5' ACGGCTACCTTGTTACGACTT 3'. Reaction was performed in mixture containing: 0.2 μM of each primer, 0.2 μg of chromosomal DNA, 250 μM of each dNTP, 1 U of DNA polymerase (*Hypernova*, DNA-Gdańsk, Poland) in 1 × PCR buffer (20 mM Tris-HCl pH 8.8, 10 mM KCl, 3.4 mM MgCl_2_, 0.15% Triton X-100). The reaction mixture was incubated for 3 min at 95°C, followed by 30 cycles at 95°C for 1 min, 55°C for 1 min, 72°C for 1.5 min, and a final incubation for 5 min at 72°C using a Mastercycler Gradient (Eppendorf, Germany). PCR product was purified from an agarose gel band using DNA Gel-Out kit (A&A Biotechnology, Poland), and cloned directionally into pCR-Blunt vector (Invitrogen). The 16S rDNA insert was sequenced using ABI 3730 xl/ABI 3700 sequencing technology (Agowa DE, Germany).

### Genomic DNA library construction

The chromosomal DNA from 32c strain cells was isolated using a Genomic DNA Prep Kit (A&A Biotechnology, Poland) according to protocol for Gram-negative bacteria. The DNA was digested using the 20 U of *Sal*I and 20 U of *Bgl*II endonucleases (Fermentas, Lithuania) for 2 hours at 37°C in 1× buffer O^+ ^(Fermentas), and 2- to 8-kb fragments were purified from a 0.8% agarose gel using the DNA Gel Out kit (A&A Biotechnology, Poland). Then DNA fragments were ligated with T4 DNA ligase (Epicentre, USA) for 1 h at 16°C into pBAD/Myc/HisA vector (Invitrogen) pre-cutted with the same restriction enzymes. *E. coli *TOP10F' cells were transformed to give the genomic library by incubation at 37°C on LA agar (10 g pepton K, 5 g yeast extract, 10 g NaCl, and 15 g agar) containing 100 μg/ml ampicillin, 1 mM IPTG and 20 μg/ml X-gal. After 12 h incubation, plates were transferred to 20°C and incubated further for 16 h. Blue colonies were taken for analysis. These *E. coli *TOP10F' cells were transformed with plasmid containing the *Arthrobacter *sp. 32c β-galactosidase gene. Plasmid DNA was extracted from these recombinant strains. The insert of the smallest recombinant plasmid (pBADmycHisALibB32c) was sequenced using ABI 3730 xl/ABI 3700 sequencing technology (Agowa DE, Germany).

### β-D-galactosidase gene amplification and cloning to bacterial expression system

Based on the known β-D-galactosidase gene sequence of *Arthrobacter *sp. 32c (GenBank Accession No. FJ609657), the specific primers for PCR amplification were designed and synthesized. The gene was amplified using two separate reactions. The first DNA fragment was amplified using the forward primer: F1Nc-β-gal C**ATG**GGCAAGCGTTTTCCAAG, and reverse primer: R32c-β-gal CCCCGTCGACTTTTCTAGA**TCA**GTCCTCCGCGATCAC (containing *Sal*I and *Xba*I recognition sites, underlined). The second DNA fragment was amplified using the forward primer: F2Nc-β-gal **G**GCAAGCGTTTTCCAAGCGG, and and reverse primer: R32c-β-gal CCCCGTCGACTTTTCTAGA**TCA**GTCCTCCGCGATCAC (containing *Sal*I recognition site, underlined). The start and stop codons are given in bold. For the NcoI sticky end generation the second forward F2Nc-β-gal primer contains only one nucleotide of the start codon. Each PCR reaction mixture contained: 0.2 μM of each primer, 0.2 μg of pBADmycHisALibB32c DNA, 250 μM of each dNTP, 1 U of DNA polymerase (*Hypernova*, DNA-Gdańsk, Poland) in 1 × PCR buffer (20 mM Tris-HCl pH 8.8, 10 mM KCl, 3.4 mM MgCl_2_, 0.15% Triton X-100). The reaction mixtures were incubated for 3 min at 95°C, followed by 5 cycles at 95°C for 1 min, 50°C for 1 min, 72°C for 2 min and 25 cycles at 95°C for 1 min, 60°C for 1 min, 72°C for 2 min and a final incubation for 5 min at 72°C using a Mastercycler Gradient (Eppendorf, Germany). Both amplification reaction products were purified and mixed together at ratio 1:1. This mixture was denaturated at 95°C for 3 min and cooled down to room temperature at 0.2°C/s. Afterwards DNA were purified by ethanol precipitation, digested with *Sal*I endonuclease and cloned into pBAD/Myc/HisA (Invitrogen) vector pre-cutted with *Nco*I and *Sal*I endonucleases. The resulting recombinant plasmid pBAD/Myc/HisA-β-gal32c containing the *Arthrobacter *sp. 32c β-D-galactosidase gene under control of the pBAD promoter was used to transform chemically competent *E. coli *LMG194 plysN cells [29]

### Expression of the recombinant β-D-galactosidase gene in *E. coli*

The recombinant plasmid pBAD/Myc/HisA-32cβ-gal was used for the expression of the putative β-D-galactosidase gene in *E. coli *LMG 194 plysN under the control of pBAD promoter. The cells were grown overnight at 37°C in LB medium containing chloramphenicol (34 μg/ml) and ampicillin (100 μg/ml) in air shaker at 220 rpm. The preculture was inoculated (1%) into fresh 1 liter of LB medium containing the same antibiotics and cultivation was continued at 37°C to OD_600 _of 0.5. The culture was then supplemented with 0.02% (w/w) arabinose (final concentrations) and grown for 4 h at 37°C to achieve the overexpression of β-D-galactosidase gene.

### *Pichia pastoris *expression plasmids construction

The primers used for amplification of the *Arthrobacter *sp. 32c β-D-galactosidase gene were: F32c-β-gal **ATG**GGCAAGCGTTTTCCAAGCGGC and R32c-β-gal CCCCGTCGAC TTTTCTAGA**TCA**GTCCTCCGCGATCAC (containing *Sal*I and *Xba*I recognition sites, underlined) (reaction A). The start and stop codons are given in bold. The second PCR reaction was performed to obtain a linear form of DNA vectors using primers: Phos-alfa-factor phos-TCTTTTCTCGAGAGATACCCCTTCTTCTTTAGCAGCAATGC and AOX1-res-insert-ATTTGAATTCTCTAGACTTAAGCTTGTTTGTAGCCTTAGACATGACTGTT CCTCAGTTCAAGTTG and pPICZαA (reaction B) or pGAPZαB (reaction C) plasmid DNA as DNA template. Each PCR reaction mixture contained: 0.2 μM of each primer, 0.2 μg of recombinant plasmid, 250 μM of each dNTP, 1 U of DNA polymerase (*Hypernova*, DNA-Gdańsk, Poland) in 1 × PCR buffer (20 mM Tris-HCl pH 8.8, 10 mM KCl, 3.4 mM MgCl_2_, 0.15% Triton X-100). Reaction A was performed using following conditions: 95°C – 3 min, (95°C – 1 min, 53°C – 1 min, 72°C – 2 min; 5 cycles), (95°C – 1 min, 65°C – 1 min, 72°C – 2 min; 25 cycles), 72°C – 5 min. Reaction B and C were performed at conditions: 95°C – 3 min, (95°C – 1.5 min, 66°C – 1 min, 72°C – 4 min; 5 cycles), (95°C – 1.5 min, 68°C – 1 min, 72°C – 4 min; 25 cycles), 72°C – 10 min. The PCR products were purified from an agarose gel bands using DNA Gel-Out kit (A&A Biotechnology, Poland), digested with *Xba*I endonuclase and ethanol precipitated. The DNA fragments from reaction A and B and from reaction A and C were ligated with each other and chemically competent *E. coli *TOP10F' (Invitrogen) cells were transformed with those ligation mixtures, spread out on LA plates containing 12.5 μg/ml zeocine (Invitrogen) and incubated at 37°C for 16 h. Afterwards recombinant plasmids were isolated, linearized by *Sac*I or *Xma*JI endonuclease and used to transform *P. pastoris *GS115 competent cells using *Pichia *EasyComp™ Transformation Kit (Invitrogen). The obtained *P. pastoris *GS115 recombinant strains harbouring pGAPZαA-32cβ-gal or pPICZαA-32cβ-gal recombinant plasmids were used to extracellular production of the *Arhrobacter *sp. 32c β-D-galactosidase.

### Expression of the β-D-galactosidase gene in *Pichia pastoris*

The *P. pastoris *GS115 recombinant strains harbouring pGAPZαA-32cβ-gal or pPICZαA-32cβ-gal plasmid were used to extracellular expression of the *Arhrobacter *sp. 32c β-D-galactosidase either constitutively or after methanol induction, respectively. For both expression systems 900 ml of YPG medium (Yeast extract 1%, Pepton K 2%, 2% glycerol) was inoculated with 100 ml of YPG medium cells cultures of the *P. pastoris *pGAPZαA-32cβ-gal or *P. pastoris *pPICZαA-32cβ-gal. In case of the constitutive β-D-galactosidase expression the inoculated culture was grown with agitation at 30°C for 4 days. After 2 days additional carbon source in form of glycerol was added to final concentration of 3% v/v to the broth. In case of the methanol induced variant, 100 ml overnight culture of the *P. pastoris *pPICZαA-32cβ-gal was centrifugated at 1500 × *g *for 10 min. The supernatant was discarded, cells were dissolved in 100 ml of BMMY medium (1% yeast extract, 2% peptone, 0.004% L-histidine, 100 mM potassium phosphate, pH 6.0, 1.34% YNB, 4 × 10^-5^% biotin, 0.5% methanol) and added to 900 ml of the same medium. The cultivation was performed for 4 days, where methanol was added to final concentration of 0.65%, 0.8% and 1% after first, second and third day, respectively.

### β-D-galactosidase purification

After protein expression in *E. coli *host, the cells were disrupted according to protocol described earlier with some modifications [29]. Cells were harvested by centrifugation at 5,000 × *g *for 20 min and the cell pellet was resuspended in 30 ml of buffer A (20 mM K_2_HPO_4_-KH_2_PO_4_, pH 7.5) and frozen at -20°C for 15 min. After thawing at room temperature, the samples were centrifuged at 10,000 × *g*. The supernatant containing the desired protein was applied onto affnity matrix of agarose coupled with p-aminobenzyl-1-thio-β-D-galactopyranoside (PABTG-agarose, Sigma) (10 ml column) equilibrated with four volumes of buffer A. The column was washed with 300 ml of the buffer A, and the recombinant β-D-galactosidase was eluted three times with 10 ml of 0.05 M sodium borate (pH 10.0) buffer at a flow rate of 0.5 ml/min. Active fractions containing the β-D-galactosidase were collected and dialyzed three times against 3 L of buffer D (100 mM NH_4_HCO_3_).

In case of the purification of the extracellular produced β-D-galactosidase in *P. pastoris *cultures, the yeast cells were separated from the post-culture medium through centrifugation. Next, the ammonium sulphate was added to the post-culture medium to 60% w/w, at 4°C. The precipitated proteins were centrifugated at 20,000 × *g*, dissolved in buffer A and dialyzed overnight against the same buffer. For β-D-galactosidase purification the dissolved sample was applied further directly onto affnity matrix of agarose coupled with p-aminobenzyl-1-thio-β-D-galactopyranoside and purified as described above for bacterial system. The concentration of purified protein was determined by the Bradford method using bovine serum albumin (BSA) as a standard.

### β-D-galactosidase activity assays

The activity of purified *Arthrobacter *sp. 32c β-D-galactosidase was determined by the use of chromogenic substrates as described elsewhere [4,14]. The *o*-nitrophenol released from 10 mM of *o*-nitrophenyl-β-D-galactopyranoside (ONPG) by β-D-galactosidase at 0–70°C and pH range 4.5–9.5 (0.02 M citrate buffer for pH 4.5 and 5.5; 0.02 M K_2_HPO_4_-KH_2_PO_4 _for pH 6.5 and 7.0 and 0.02 M Tris-HCl for pH 8.5 and 9.5) was measured at 405 nm. The reaction was stopped after 10 min with 1 M Na_2_CO_3_. One unit is defined as one micromolar of *o-*nitrophenol released per minute.

Substrate specificity was estimated using 1 mM solution of chromogenic substrates: *o*-nitrophenyl-β-D-galactopyranoside (ONPG), *p*-nitrophenyl-β-D-galactopyranoside (PNPG), *o*-nitrophenyl-β-D-glucopyranoside (ONPGlu) and *p*-nitrophenyl-β-D-glucopyranoside (PNPGlu). Activity determination was carried out under standard conditions in 0.02 M K_2_HPO_4_-KH_2_PO_4 _(pH 6.5) buffer at 10, 20, 30, 40 or 50°C. The activity of the β-D-galactosidase towards lactose was monitored by HPLC analysis (column Bio-rad, Aminex HPX-87H) where 1% solutions of lactose, glucose, fructose and galactose were used as standards.

In the combined enzyme assay glucose isomerase from *Streptomyces murinus *(Sigma G4166) was used in the amount of 0.01 g/ml of 5% w/v solution of lactose (0.02 M K_2_HPO_4_-KH_2_PO_4_, pH 6.5). The *Arthrobacter *sp. 32c β-D-galactosidase was used at concentration of 200 U/ml of the mixture. The reaction mixture was set at 37°C for 72 h and products were analysed by HPLC every 12 hours.

Effects of 5 mM dithiothreitol, 5 mM of 2-mercaptoethanol, 5 mM of L-cysteine, 5 mM of reduced glutathione, and metal ions (Na^+^, K^+^, Mn^2+^, Mg^2+^, Ca^2+^, Fe^2+^, Zn^2+^, Cu^2+^, Co^2+ ^and Ni^2+^; each at concentration of 5 mM) on *Arthrobacter *sp. 32c β-D-galactosidase activity were determined under standard conditions.

All measurements and/or experiments were conducted five times. Results are presented as mean SD. Relative activities were estimated in above experiments by comparison to highest activity (100%).

## Authors' contributions

PH carried out the molecular genetic studies, participated in the design of the study and drafted the manuscript. MW carried out the molecular genetic studies, participated in drafted the manuscript. JK conceived of the study, and participated in its design and coordination. All authors read and approved the final manuscript.
